# When virtual and real worlds coexist: Visualization and visual system affect spatial performance in augmented reality

**DOI:** 10.1167/jov.21.8.17

**Published:** 2021-08-13

**Authors:** Tatjana Pladere, Artis Luguzis, Roberts Zabels, Rendijs Smukulis, Viktorija Barkovska, Linda Krauze, Vita Konosonoka, Aiga Svede, Gunta Krumina

**Affiliations:** 1 Department of Optometry and Vision Science, Faculty of Physics, Mathematics and Optometry, University of Latvia, Riga, Latvia; 2 Laboratory of Statistical Research and Data Analysis, Faculty of Physics, Mathematics and Optometry, University of Latvia, Riga, Latvia; 3 LightSpace Technologies, Marupe, Latvia

**Keywords:** augmented reality, depth cues, perceptual matching, head-mounted display, binocular and accommodative disorders

## Abstract

New visualization approaches are being actively developed aiming to mitigate the effect of vergence-accommodation conflict in stereoscopic augmented reality; however, high interindividual variability in spatial performance makes it difficult to predict user gain. To address this issue, we investigated the effects of consistent and inconsistent binocular and focus cues on perceptual matching in the stereoscopic environment of augmented reality using a head-mounted display that was driven in multifocal and single focal plane modes. Participants matched the distance of a real object with images projected at three viewing distances, concordant with the display focal planes when driven in the multifocal mode. As a result, consistency of depth cues facilitated faster perceptual judgments on spatial relations. Moreover, the individuals with mild binocular and accommodative disorders benefited from the visualization of information on the focal planes corresponding to image planes more than individuals with normal vision, which was reflected in performance accuracy. Because symptoms and complaints may be absent when the functionality of the sensorimotor system is reduced, the results indicate the need for a detailed assessment of visual functions in research on spatial performance. This study highlights that the development of a visualization system that reduces visual stress and improves user performance should be a priority for the successful implementation of augmented reality displays.

## Introduction

### Overview

Recent developments in visualization technologies for augmented reality have led to a growing interest toward spatial perception research that aims to discover the potential benefits and limitations of new displays intended for use in professional capacities. Precise perception and the interpretation of digital information are crucial for decision making and task performance in many areas, such as healthcare, education, aerospace, manufacturing, and defense (Gorbunov, 2014; Kang, Azizian, Wilson, Wu, Martin, Kane, Peters, Cleary, & Shekhar, 2014; Douglas, Wilke, Gibson, Boone, & Wintermark, 2017; Eckert, Volmerg, & Friedrich, 2019; Uppot, Laguna, McCarthy, De Novi, Phelps, Siegel, & Courtier, 2019).

From a technical standpoint, for augmented digital overlays to have a meaningful contribution, the content has to provide information about image location that would be concordant with the physical environment. However, a single focal plane in the typical stereoscopic display is a technological limitation that makes mimicking natural-viewing condition impossible. The stereoscopic display renders two separate images of a scene, one for each eye. To perceive them as a single binocular image, disparity-driven vergence eye movements align the two visual axes, and the visual system fuses images and creates a sense of depth. Although vergence distance varies depending on the disparity of rendered images, the focal distance remains fixed all the time. Thus, display's inability to produce accurate focus cues at different viewing distances is a problem for the visual system to solve because it disturbs the normal coupling of vergence and accommodation. The resultant conflicts between binocular and focus cues can be associated with discrepancies in spatial perception (Condino, Carbone, Piazza, Ferrari, & Ferrari, 2020; Peillard, Argelaguet, Normand, Lécuyer, & Moreau, 2020). New visualization approaches, such as multifocal, varifocal, and holographic displays, aim to mitigate or eliminate this issue (Rolland, Krueger, & Goon, 2000; Huang & Hua, 2018; Zabels, Osmanis, Narels, Gertners, Ozols, Rutenbergs, & Osmanis, 2019; Zhan, Xiong, Zou, & Wu, 2020). Nevertheless, the actual user gain remains difficult to predict due to high interindividual variability and lack of agreement in perceptual studies on whether consistency of binocular and focus cues is a mandatory requirement for accurate spatial judgments in augmented reality (Watt, Akeley, Ernst, & Banks, 2005; Hoffman, Girshick, Akeley, & Banks, 2008; Naceri, Chellali, & Hoinville, 2011; Peillard et al., 2019; Erkelens & MacKenzie, 2020; Peillard, Itoh, Normand, Argelaguet, Moreau, & Lecuer, 2020; Gao, Peillard, Normand, Moreau, Liu, & Wang, 2020). Here, we describe how the consistency of binocular and focus cues impacts distance matching between physical objects and images in stereoscopic augmented reality, and how useful vision screening may be for predicting the extent to which the user would benefit from the implementation of new technology. We also discuss the implications for vision research and perception-driven optimization of augmented reality displays.

### Cues for spatial performance

The three-dimensional spatial layout of objects and images is judged based on multiple information sources – depth cues. From the perspective of designing vision-friendly and viable augmented reality displays, providing consistent cues is one of the major challenges to be solved. Binocular cues (disparity and vergence) are required for the precise discrimination of the relative depth of elements in near space (Hibbard, Haines, & Hornsey, 2017; Rogers, 2019). From all available monocular cues, the focus cues (accommodation and blur in the retinal image) are considered the most linked to the binocular cues (Howard & Rogers, 2002). However, the understanding of depth is not provided by the disparity (Mon-Williams, Tresilian, & Roberts, 2000), vergence (Linton, 2020), accommodation (Ritter, 1977; Rogers, 2019; Linton, 2020), or image blur (Mather & Smith, 2002; Langer & Siciliano, 2015) alone. Therefore, it is important to understand how different signals are combined to form a unified representation of the spatial layout.

Combining multiple sources of commensurate information is required to derive a percept of three-dimensional location (Svarverud, Gilson, & Glennerster, 2010). Models explaining the combination of depth cues have been strongly debated (Landy, Maloney, Johnston, & Young, 1995; Jacobs, 2002; Tyler, 2020) and revealed the importance of cue reliability. According to Bayesian theories of statistically optimal cue combination (Landy et al., 1995; Tyler, 2020), the information is summed up or processed in a selective way depending on the visual context (Howard & Rogers, 2002; Sweet, Kaiser, & Davis, 2003). In a Bayesian model, perceptual estimates take the form of probability distributions, rather than determinate values (Jacobs, 2002). Therefore, the available cues are combined in a flexible manner according to their weights, which are proportional to the inverse variances of the cue distributions.

In natural viewing, all cues are available and provide consistent depth information. However, display images may contain limited, imprecise, and contradictory depth cues. As a result, the conflicts between different signals occur and the visual system has to solve them. If the conflict between cues is large, the visual system usually exhibits cue vetoing. In the case of cue vetoing, spatial judgments are determined by one depth cue, with the other cue being suppressed (Sweet, Kaiser, & Davis, 2003; Tyler, 2020). Some evidence has also been provided for the possibility of cue switching meaning that perceptual judgments were based on different cues, the contribution of which was time-multiplexed (Van Ee, van Dam, & Erkelens, 2002). However, if the conflict is decreased, depth perception is based on a weighted linear combination of the available cues (Landy et al., 1995; Jacobs, 2002) with a dominant cue being promoted for the accelerated processing (Tyler, 2020). It should be noted that the combination of cues can vary considerably on an individual level (Girshick & Banks, 2009; Wismeijer, Erkelens, van Ee, & Wexler, 2010), possibly explaining variations in the accuracy of spatial judgments in natural viewing (Todd & Norman, 2003; Norman, Adkins, & Pederson, 2016). In general, the availability and consistency of depth cues plays a crucial role in the accuracy of perceptual judgments and task completion time (Mather & Smith, 2004).

### Binocular and focus cues in augmented reality

An augmented reality display should render images with concordant depth cues in order to ensure a smooth and successful merger of virtual and real worlds. However, most conventional displays are unable to provide consistent binocular and focus cues at different viewing distances. Specifically, if only one focal plane is used in the stereoscopic head-mounted display, the eyes should accommodate on the focal plane and converge at the stereoscopic scene depth (Howarth, 2011). This decoupling may be attributed to user discomfort (Hoffman et al., 2008; Shibata, Kim, Hoffman, & Banks, 2011; Koulieris, Bui, Banks, & Drettakis, 2017).

As the conflict between binocular and focus cues has been identified as a paramount issue affecting user comfort, the alternative approaches for display architectures have been developed to mitigate or eliminate it. In particular, several studies provided some theoretical and experimental support for the implementation of multiple planes in the architecture of the display's optical element (Rolland, Krueger, & Goon, 1999; Akeley, Watt, Girshick, & Banks, 2004; Watt et al., 2005; Hoffman et al., 2008, MacKenzie, Hoffman, & Watt, 2010; Shibata et al., 2011), leading to a growing interest toward the practical implementation of this approach in augmented reality headsets (Rolland, Krueger & Goon, 2000; Love, Hoffman, Hands, Gao, Kirby, & Banks, 2009; Hu & Hua, 2014; Chang, Kumar, & Sankaranarayanan, 2018; Huang & Hua, 2018; Zabels et al., 2019; Zhan et al., 2020). The key underlying idea is to use several distinct image planes in order to minimize the magnitude of the conflict between binocular and focus cues, thus covering a wider range of distances for comfortable viewing. It can be achieved in different ways, for instance, by using beam splitters to superimpose images (Akeley et al., 2004) or high-speed switchable lenses to change the optical distance of the image plane (Love et al., 2009). Despite the availability of such displays, there is still no clear evidence that they have a positive impact on user performance in terms of spatial perception in comparison to conventional visualization systems (Peillard et al., 2020) due to a lack of corresponding research and the amount of controversy in the rapidly developing understanding of human factors.

The role of depth cues together with factors underlying high individual variations in spatial judgments are currently the subject of prolonged debates leading to limitations in making predictions about the usefulness of new augmented reality displays. Three themes in particular have emerged from these discussions:

1. Consistent binocular and focus cues as prerequisites of accurate spatial judgments. At first, focus cues were not considered important due to a high variability of accommodative responses unrelated to spatial judgments (Baird, 1903; Ritter, 1977; Owens & Liebowitz, 1980; Durgin, Proffitt, Olson, & Reinke, 1995; Mon-Williams, Tresilian, & Roberts, 2000) and specifics of a blur cue (it does not indicate the direction of a change in viewing distance). The stereoscopic way of showing information used to be a common approach in front-view and head-mounted displays. However, the situation changed when the appropriate focus cues were called a key determinant of precise and fast spatial judgments (Akeley et al., 2004; Watt et al., 2005; Hoffman et al., 2008). Since then, the mismatch between binocular and focus cues has been frequently used as an explanation of different discrepancies in perceptual judgments (Watt et al., 2005; Hoffman et al., 2008; Froner, 2011; Naceri, Moscatelli, & Chellali, 2015; Vienne, Plantier, Neveu, & Priot, 2018; Lin, Caesaron & Woldegiorgis, 2019), as well as an essential constituent of the driving force for the development of alternative displays. However, recent experimental findings appear to challenge this suggestion. For instance, spatial judgments were not compromised when the concordant focus cues were lacking due to the technical aspects of the display, viewing condition, or age-related specifics of vision (Singh, Ellis, & Swan, 2018; Condino et al., 2020; Peillard et al., 2020). Images with conflicting binocular and focus cues were considered clearer than digital content presented without the cues conflict (Erkelens & MacKenzie, 2020). Adding a consistent accommodation cue to an image even resulted in impaired performance for some individuals (Linton, 2020). Consequently, the importance of appropriate focus cues for precise spatial judgments in the binocular viewing of digital information remains questionable (Langer & Siciliano, 2015; Maiello, Chessa, Solari, & Bex, 2015; Schmidt, Bruder, & Steinicke, 2017; Rogers, 2019; Linton, 2020; Peillard et al., 2020).
2. Overestimation because of a mismatch between binocular and focus cues in near space. In the theoretical framework, perceptual distances were commonly expected to be biased toward the focal plane, and therefore overestimated in the stereoscopic environment when the images were displayed in front of a focal plane (Drascic & Milgram, 1996; Peli, 1999). These predictions were supported in a number of experimental studies (Swan, Singh, & Ellis, 2015; Lin, & Woldegiorgis, 2017; Lin, Caesaron, & Woldegiorgis, 2019). However, it was also shown that individuals both underestimated and overestimated distances under viewing conditions with constant vergence-accommodation conflicts (Napieralski, Altenhoff, Bertrand, Long, Babu, Pagano, Lern, & Davis, 2011; Rousset, Bourdin, Goulon, Monnoyer, & Vercher, 2018, Peillard et al., 2020). Moreover, the matched distances could be veridical (or close to veridical) in the presence of conflict, the magnitude of which varied from around 0.5 D to 4.0 D (Naceri, Chellali, & Hoinville, 2011; Shibata et al., 2011; Vienne et al., 2018; Vienne, Masfrand, Bourdin, & Vercher, 2020).
3. Visual factors underlying high interindividual variability. Some individuals make accurate judgments even in the presence of conflicting cues, whereas the performance of others is affected negatively by it (Watt et al., 2005; Hoffman et al., 2008; Naceri, Chellali, & Hoinville, 2011; Vienne et al., 2018). In search for plausible explanations, some attempts were made to find associations between the behavioral results and visual functions (Watt et al., 2005; Hoffman et al., 2008; McIntire, Wright, Harrington, Havig, Watamaniuk, & Heft, 2014; Erkelens & MacKenzie, 2020), however, no strong correlations were observed, possibly due to a limited number of participants (from 4 to 12 participants in the above-mentioned studies). As a result, the understanding of the genesis of individual performance variations remains drastically incomplete.

Altogether, it is clear that the perceptual mismatch cannot be explained by the vergence-accommodation conflict due to the visualization method alone. Even if the binocular and focus cues in augmented reality indicate the same information about depth of image, it does not mean that they will be perceived as the same.

The availability and reliability of depth cues in augmented reality are often assessed from the perspective of digital stimulus. However, these parameters depend not only on the type of visualization, but also on the capability of the human visual system to react to the provided signals and tolerate visual stress. Generally, the visual system can tolerate some discrepancy between stimuli. However, the stress test induced by stereoscopic images with conflicting binocular and focus cues can be especially challenging for individuals with binocular and accommodative disorders. Most often individuals accommodate or converge less than required, which may affect spatial judgments in the presence of vergence-accommodation conflict due to recalibrated weights of the depth cues (Horwood & Riddell, 2014). The individuals are often unaware of binocular and accommodative disorders due to the absence of symptoms (Horwood & Riddell, 2008; Chandra & Akon, 2016; Atiya, Hussaindeen, Kasturirangan, Ramasubramanian, Swathi, & Swaminathan, 2020). Moreover, visual acuity and stereoscopic acuity, commonly used as the inclusion criteria in the studies on human factors in stereoscopic augmented reality, can be according to clinical norms (Scheiman & Wick, 2013). Consequently, there is a chance that the previous studies on perceptual matching in augmented reality with vergence-accommodation conflict included individuals with binocular and accommodative disorders along with individuals who had normal vision.

### Rationale of the present study

Taken together, previous research suggests that spatial perception in stereoscopic augmented reality can be influenced not only by the technical realization of visualization, but also by individual variations in vision possibly affecting the weights of binocular and focus cues. Despite the increasing interest in the research on human factors, there are limited data available that would allow the prediction of user acceptance of new displays, explain the variations in the results of behavioral studies, and assess the possible user gain in the future. For this reason, we would like to contribute to the ongoing formation of understanding by demonstrating the impact of vision on spatial perception when the augmentation of reality is ensured in different ways. To assess the effect of consistency of image planes and display focal planes (resulting in consistency of provided binocular and focus cues) on perceptual distance matching in stereoscopic augmented reality, we used a headset prototype with multifocal architecture. By driving it in the single focal plane mode, the vergence-accommodation conflict was induced. Here, we investigate how individuals with normal vision and those with mild binocular and accommodative disorders can accomplish perceptual distance matching in the environment of stereoscopic augmented reality under consistent-cues and inconsistent-cues condition.

## Method

### Participants

A total of 58 healthy participants (19 men and 39 women) volunteered to participate in the study. Participants’ ages ranged from 20 to 30 years. Visual functions of each participant were tested before completing the task. The inclusion criteria were as follows: normal or corrected-to-normal (with contact lenses) visual acuity, stereoacuity of 60 arcsec or better, no signs of amblyopia, anisometropia, or strabismus, no organic findings in the eyes, no neurological findings, and no symptomatic complaints. Participants reported limited or no prior familiarity with head-mounted displays. We analyzed data of 40 participants, excluding 18 participants who did not complete the experiment due to the following reasons: diplopia (2), monocular suppression (4), inability to match the distance within 240 cm range of the linear stage (6), and failed calibration (6).

The study was approved by the Ethics Committee of the University of Latvia. It was conducted in accordance with principles of the Declaration of Helsinki.

### Assessment of visual functions

A thorough vision screening was performed before the experiment. Specifically, monocular and binocular visual acuities were tested at 40 cm distance and 5 m distance using a Snellen chart. Near stereoacuity thresholds (at 40 cm viewing distance) were measured using a near stereopsis vision test (Titmus stereo test; Stereo Optical Co., Chicago, IL, USA). Vergence (with 8Δ base in/8Δ base out vergence flipper) and binocular accommodative (with ±2.00 D lens flipper) facility was measured at 40 cm over the course of 60 seconds. Subjective break and recovery point of convergence were measured using the push up test while a participant was fixating on a single letter that corresponded to 0.2 logMAR visual acuity. The type and magnitude of far (5 m) and near (40 cm) phoria were verified using the cover test and alternating prism cover test. Convergent and divergent fusional reserves were measured using a prism bar while participants viewed a single line of vertical text that corresponded to 0.2 logMAR visual acuity.

The obtained results of visual functions were evaluated corresponding to the clinical norms defined by Scheiman and Wick (2013). To meet the definition of non-strabismic binocular and accommodative disorders, at least two visual functions were determined as not fitting the clinical norms.

### Apparatus

**Figure 1.**
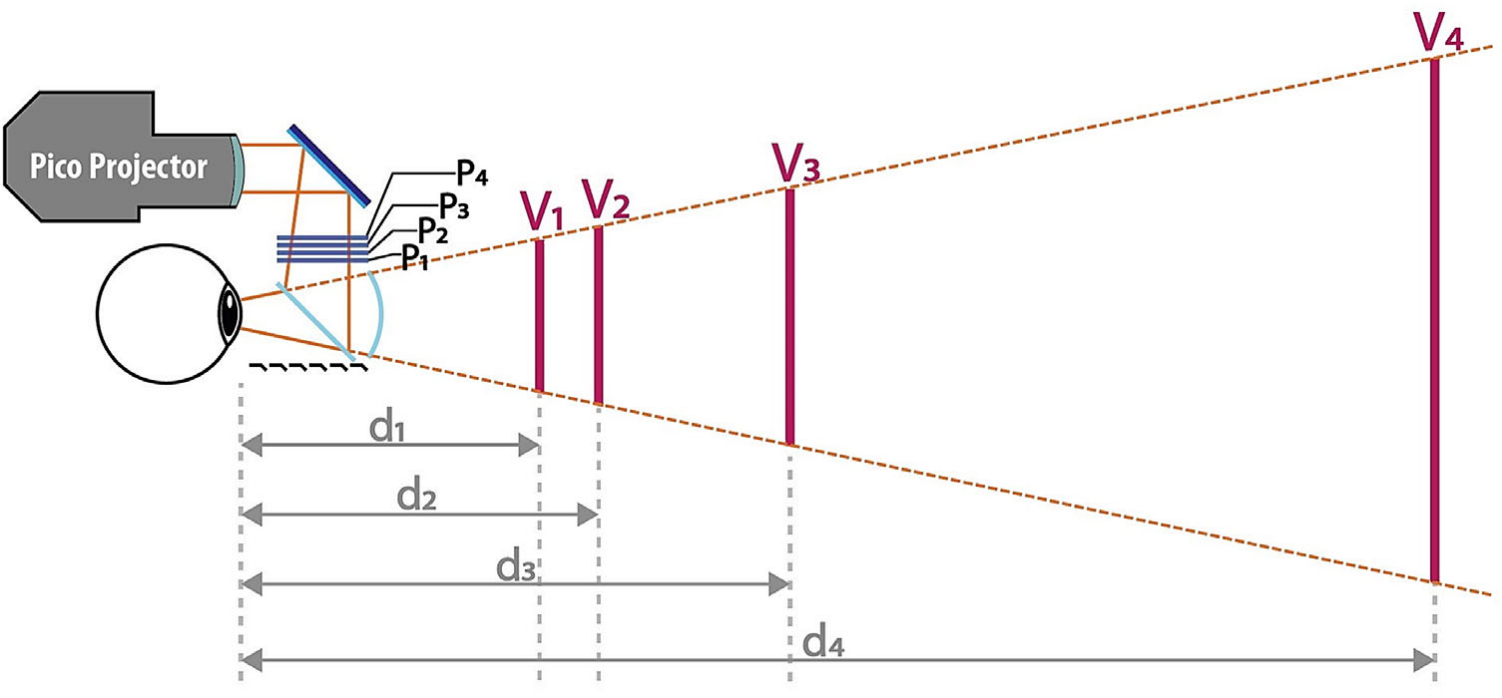
Schematic illustration of the augmented reality headset prototype used in the study. The pico- projection unit projects image frames onto the physical optical diffuser elements *p*_1_, *p*_2_, *p*_3_, and *p*_4_, which are activated in a time-multiplexed manner. The viewer when looking at the physical screens through a magnifying eyepiece sees them as virtual image planes *V*_1_, *V*_2_, *V*_3_, and *V*_4_ located at distances *d*_1_, *d*_2_, *d*_3_, and *d*_4_, respectively.

Images were presented using a LightSpace Technologies IG-1005 prototype headset (Zabels et al., 2019). It is a stereoscopic augmented reality display device utilizing stacked switchable optical diffuser elements (liquid-crystal diffusers) to physically separate display planes (*p*_1_-*p*_4_). A “bird-bath” optical image combiner is used to magnify the images formed on diffuser elements by a rear image projector ensuring four focal planes (*V*_1_-*V*_4_) optically located at *d*_1_ = 45 cm (2.22 D), *d*_2_ = 65 cm or (1.54 D), *d*_3_ = 115 cm (0.87 D), and *d*_4_ = 530 cm (0.19 D; see Figure 1).

In operation, diffuser elements are driven between a highly light transparent state and a highly light scattering state (screen mode). In the transparent state, the diffuser elements allow more than 95% of visible light to pass. Thus, the images from deeper layers are not affected by other diffuser elements in any noticeable way, and the focal planes are identical from the standpoint of image metrics.

In this architecture, the image source is a miniature high refresh-rate projection unit, outputting image depth planes sequentially in time. As seen in Figure 1, the output from the pico-projection unit is folded by a full mirror, then it illuminates stacked optical diffuser elements. The “bird-bath” optics or optical image combiner, which is formed by one flat 50/50 beam splitter and one aspherical 50/50 beam splitter, is used for the combination and magnification of images. Although it comes at a cost of reduced ambient light throughput, the reflective magnifying optics ensures a high image quality with well-controlled chromatic aberrations. The headset interfaces with a host computing platform through a wired DisplayPort connection.

### Study design

**Figure 2.**
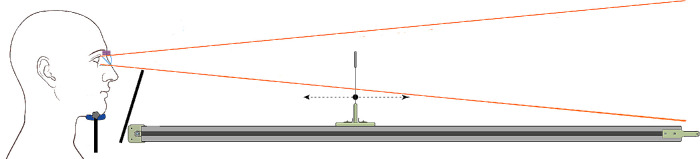
Schematic side view of the experimental setup. The participant with a headset sat in front of the linear stage (total length – 240 cm). The adjustable-height occluding surface was used to block the view of the linear stage. The linear stage was equipped with a sliding carriage that held a physical pointer on the top of a thin pole. The sliding carriage could be moved in two directions – closer and further away from the observer.

The experimental setup consisted of a motorized linear stage with a sliding carriage (see Figure 2). A thin metal pole was mounted on the top of the carriage, whereas a physical object (pointer) was mounted on the top of the pole. The participant could move the pointer in two directions – closer and further away, with a help of a controller (max speed: 5 cm/s). Given the mechanical characteristics of the system, the pointer could be positioned with a precision of 1 mm. The participant sat facing the linear stage and wore the headset. The seating height was varied using an adjustable chair. To ensure a uniform viewing angle and minimize the possible effect of head motion on perceptual judgments, participants rested their chins on a chinrest fixed on the tabletop. The fixation of head position and adjustable-height occluding surface were required to ensure that the participant did not see the rail and could not use its appearance as an additional depth cue.

Before the task, each participant underwent the display calibration procedure. First, the interpupillary distance of each participant was determined. Then, this value was used for the image rendering engine – as the rendering parameter. To fine-tune the alignment in software, a calibration image was shown through the headset to the participant on each focal plane separately. In consonance with the output of the calibration image, the physical stimulus on the linear stage was set to the corresponding distance of the given focal plane. Similar to the procedure implemented in Livingston, Ellis, White, Feiner, and Lederer (2006), the participant was asked to adjust the digital image offset for two parts of a calibration image while looking at the physical stimulus. The adjustments were performed until the participant saw the calibration image as a symmetrical cross. The calibration steps were repeated two times for all focal planes to test the consistency and accuracy of the obtained results. Careful calibration allowed us to present visual stimuli with the intended vergence and focal distance accurately while keeping visual angle constant along the line of sight.

Next, the perceptual distance matching task followed. The variability in the depth cues was achieved by switching between a multifocal (consistent-cues condition) and single focal plane mode (inconsistent-cues condition), when deactivating all but one display plane. Thus, both conditions were realized using the same headset – ensuring the identical attributes of the conveyed images (i.e. the field of view, image brightness, image refresh rate, and color balance).

In both conditions, the vergence stimuli varied corresponding to the image demonstration distance. However, the focal stimulus was equal to the vergence stimulus in the consistent-cues condition, and fixed – in the inconsistent-cues condition. The image was demonstrated at three distances from the participant: 45 cm, 65 cm, and 115 cm, which corresponded to 2.22 D, 1.54 D, and 0.87 D demand, respectively. These rendered image distances were chosen in order to match the distances of focal planes when the display was driven in the multifocal mode. In the consistent-cues condition, the images were displayed at the focal distances of planes that coincided with the rendered image distances. In the inconsistent-cues condition, only the display plane with the focal distance at 530 cm (0.19 D) was used. The induced conflict magnitude (*c*) in the stimuli to vergence and accommodation was calculated as follows: *c* = 1/ *d_v_* – 1/ *d_a_*, where *d_v_* is the rendered image distance, and *d_a_* is the focal plane distance. As a result, the conflict magnitude was 2.03 D, 1.35 D, or 0.68 D depending on the rendered image distance when the display was driven in the single focal plane mode. Trials were blocked by the condition of cues consistency. The order of the conditions was counterbalanced across participants.

The initial session included two repetitions of tasks per rendered image distance to familiarize the participants with the visual stimulus, task, and setup. Then, the experiment session followed.

The participant was shown a separate image for each eye using the headset. Provided that the fusional reserves ensured proper merging of two images, the participant saw a single image with one star in the center of a rectangular arch, and circles at the corners of it. If stereoscopic fusion failed, the participant experienced diplopia. Participants were asked to inform the experimenter immediately about the double image. In this case, the experiment was terminated. The contours of all visual stimuli were white. To avoid the potential effect of monocular suppression on spatial judgments in augmented reality (Rosales, Pointon, Adams, Stefanucci, Creem-Regehr, Thompson, & Bodenheimer, 2019), different circles were demonstrated separately to each eye. The maximum number of circles was four (in total for both eyes). The total number of circles at the beginning of the task was chosen in a random order (from 2 to 4). The possible locations were as follows: in the upper right corner, in the upper left corner, in the lower right corner, and in the lower left corner. The participant was asked to inform the experimenter immediately if the circle(s) disappeared during the trial.

In the beginning of each trial, if the participant saw one star and one arch, they responded about the number of circles perceived in the trial. The time countdown began when the response was submitted. The experiment was not time-constrained; however, the participants were instructed to complete the task as accurately and quickly as possible. The participant moved the pointer to align it with the apparent position of the projected star using a controller. When the participant finished the alignment, they reported it and closed their eyes until the next instruction. As soon as the response was given, the time countdown was stopped, and the value of matched distance was collected. Next, the experimenter changed the position of the physical pointer to one of the predefined initial distances (±5, ±10, ±15, and ±20 cm from the rendered image distance), the sequence of which randomly varied among trials, rendered image distances, and cues consistency conditions. Then, the experimenter switched on the next trial, asked the participant to open their eyes, and the next trial took place. Eight repetitions of the perceptual matching task were performed at each rendered image distance. Each participant completed 2 (cues consistency conditions) × 3 (rendered image distances) × 8 (repetitions) = 48 trials of perceptual distance matching, and the experiment yielded a total of 40 (participants) × 48 (trials) = 1920 trials in the analysis.

## Results

All participants were divided into two groups based on the results of vision screening – with normal vision (*n* = 16) and with mild binocular and accommodative disorders (*n* = 24). As revealed by statistical analysis of data (see Appendix for details), participants with mild binocular and accommodative disorders had smaller accommodative facility and vergence facility, as well as larger amounts of near and distance horizontal phorias, however, near points of convergence were similar. Participants with normal vision had larger convergent and divergent fusional reserves both at near and far.

**Figure 3.**
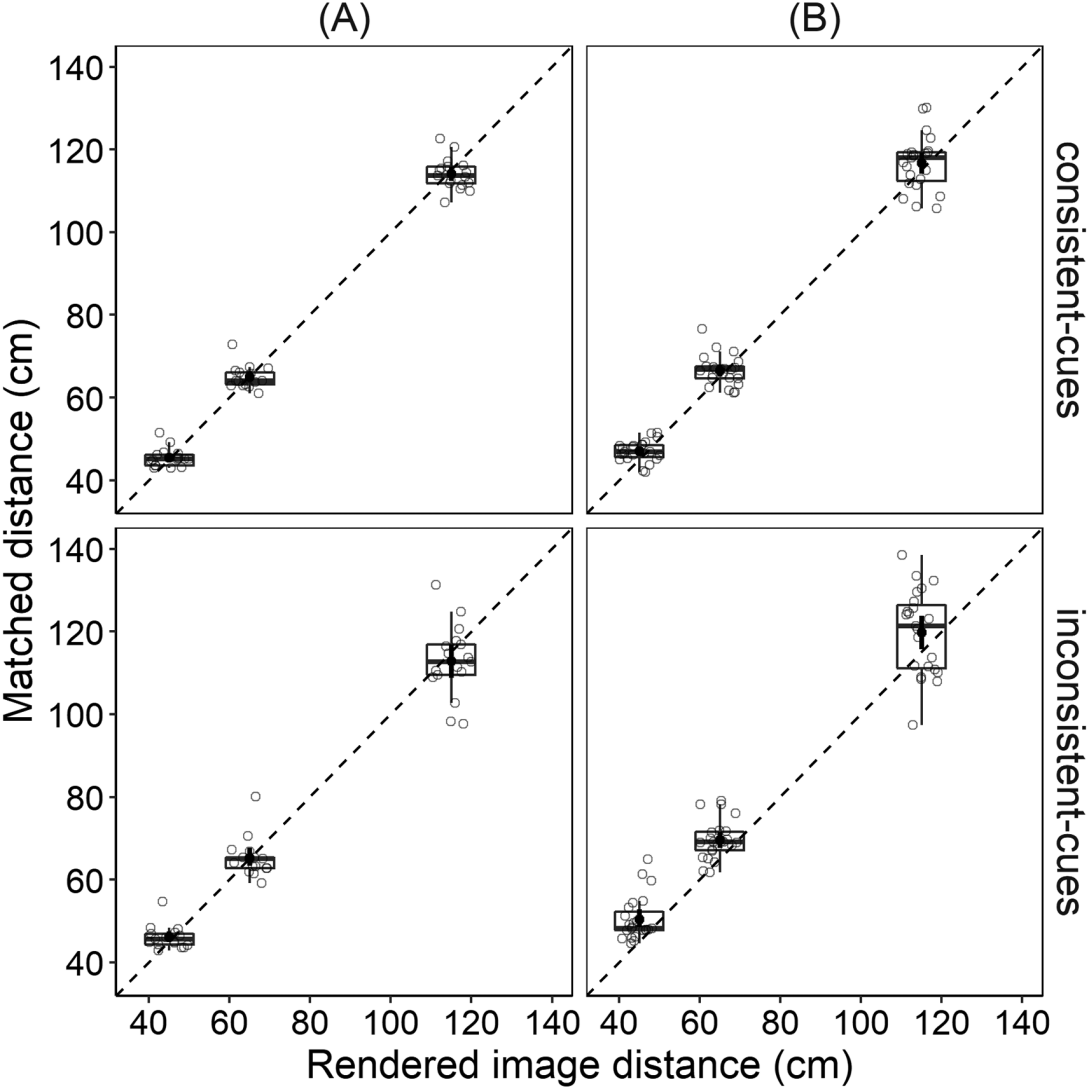
Matched distance as a function of rendered image distance in the consistent-cues condition (upper row) and inconsistent-cues condition (lower row). The left column of the figure shows the data of individuals with normal vision (**A**). The right column shows the data of individuals with mild binocular and accommodative disorders (**B**). Black dots and error bars indicate means and 95% confidence intervals (CIs), respectively. Each grey dot represents each individual's average of eight trials. The dashed diagonal lines represent veridical performance with respect to changes in rendered image distance.

We were interested in determining whether individuals with normal vision and those with mild binocular and accommodative disorders could accurately perceive spatial relations between augmented reality images and real objects when images were projected using two different projection modes of the same headset. Participants judged image distances by matching spatial position of the physical pointer with that of the displayed image. The matched distances were determined, and a mean score from eight trials in each combination of cues consistency condition × rendered image distance was computed. Figure 3 plots matched distance as a function of rendered image distance. The left half of the figure shows data from individuals with normal vision, and the right half – from individuals with mild binocular and accommodative disorders. Different rows show data from different conditions of cues consistency. If perceptual distance matching were done without errors, the data would lie on the dashed diagonal lines. Examination of Figure 3 indicates that participants with normal vision matched distances more accurately than those with mild binocular and accommodative disorders, especially in the inconsistent-cues condition.

**Figure 4.**
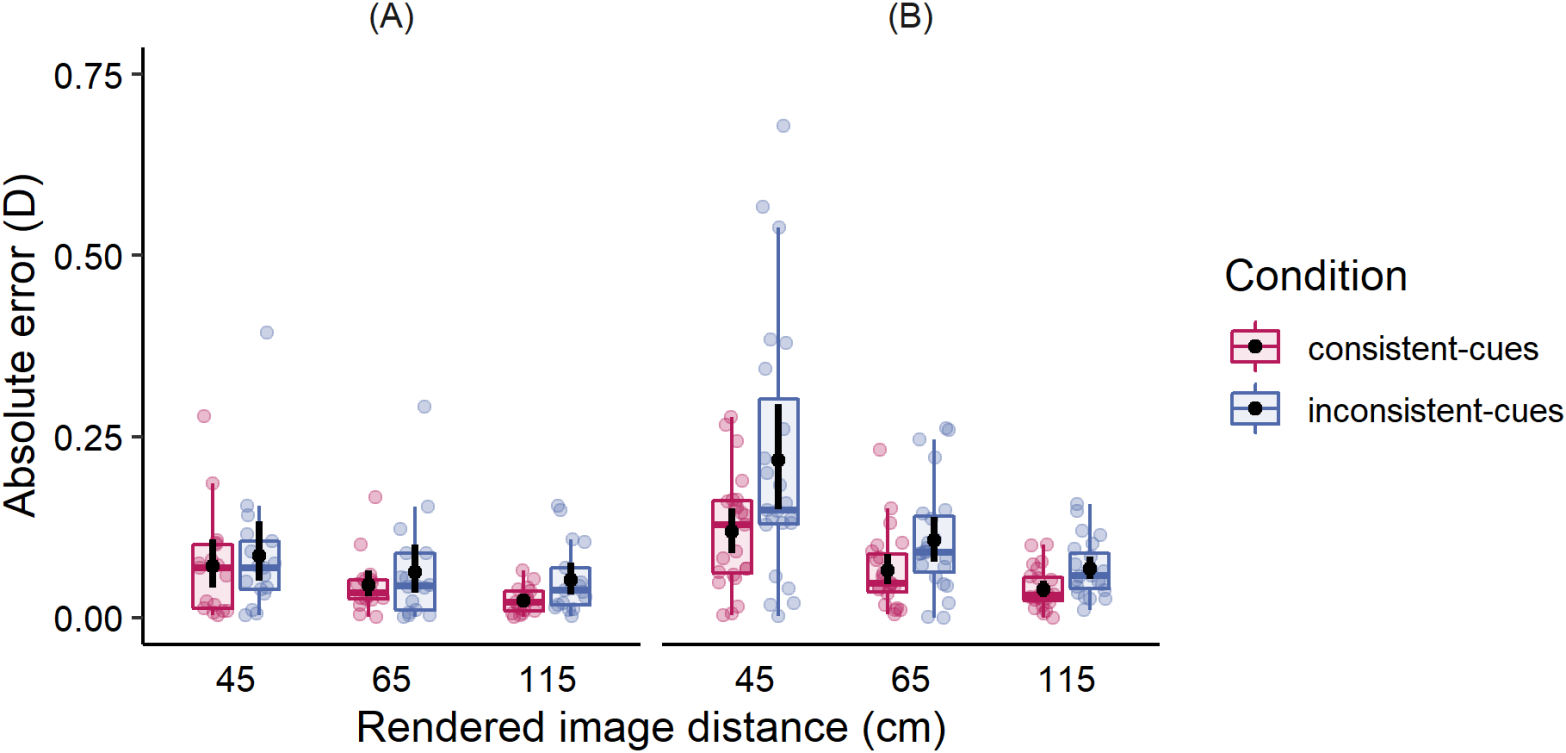
Mean magnitudes of absolute errors in the consistent-cues condition (pink symbols) and inconsistent-cues condition (blue symbols) in two groups: (**A**) with normal vision, and (**B**) with mild binocular and accommodative disorders. Black dots and error bars indicate means and 95% confidence intervals (CIs), respectively. Each color dot represents each individual's average of eight trials.

To assess the magnitude and direction of mismatch, we further analyzed the absolute errors and signed errors, respectively. For a direct comparison of distributions of errors across viewing distances, the errors were calculated in diopters. Figure 4 shows the results for the absolute errors in the consistent-cues condition (pink symbols) and inconsistent-cues condition (blue symbols). The examination of Figure 4 reveals that the consistency of binocular and focus cues led to an improved accuracy of distance matching in augmented reality. Although the reliable effect was observed in both groups, the mean benefit of cues consistency was larger for individuals with mild binocular and accommodative disorders. Regarding changes in spatial location of the projected image, the absolute errors decreased with an increase of viewing distance.

**Figure 5.**
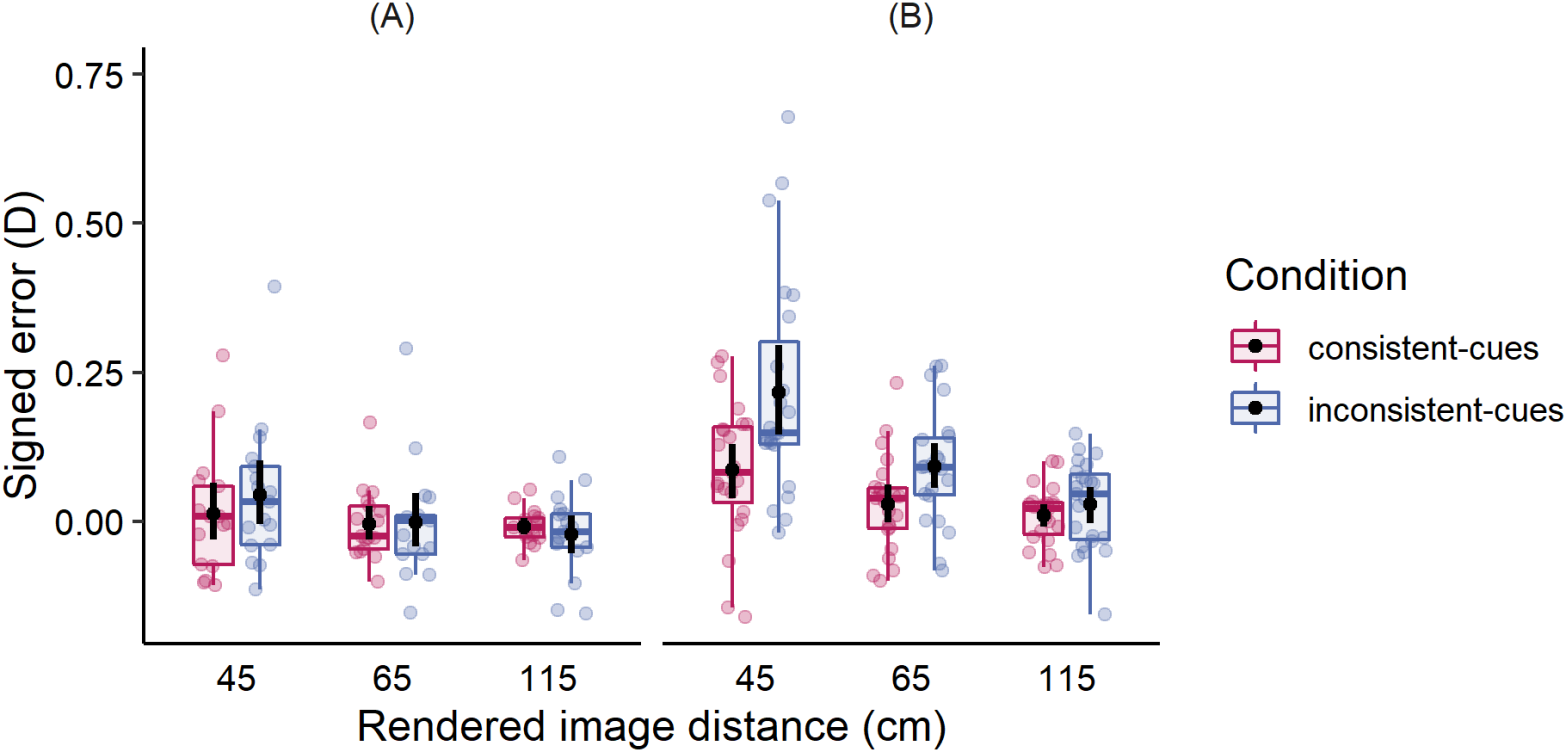
Mean magnitudes of signed errors in the consistent-cues condition (pink symbols) and inconsistent-cues condition (blue symbols) in two groups: (**A**) with normal vision, and (**B**) with mild binocular and accommodative disorders. Black dots and error bars indicate means and 95% confidence intervals (CIs), respectively. Each color dot represents each individual's average of eight trials.

To dig deeper into the specifics of mismatch, the signed errors were analyzed in addition to the absolute errors. The signed errors were classified into two groups depending on their values. Negative values occurred when the matched distance preceded the rendered image distance (interpreted as underestimation of the distance), and positive values – when the matched distance was larger than the rendered image distance (interpreted as an overestimation). Thus, comparing distributions of signed errors across different groups allowed us to investigate whether the error distribution was shifted toward positive or negative direction, which in turn implied different overestimation and underestimation patterns across experimental conditions and groups. The corresponding results are depicted in Figure 5, which shows the signed errors at three rendered image distances in both groups of participants. Image distances were both underestimated and overestimated. What is notable from Figure 5 is that the signed error distribution was clearly shifted toward overestimation in participants with mild binocular and accommodative disorders at close viewing distances. The shift was most evident when depth cues were in conflict. Participants with normal vision overestimated distances to a lower extent.

**Figure 6.**
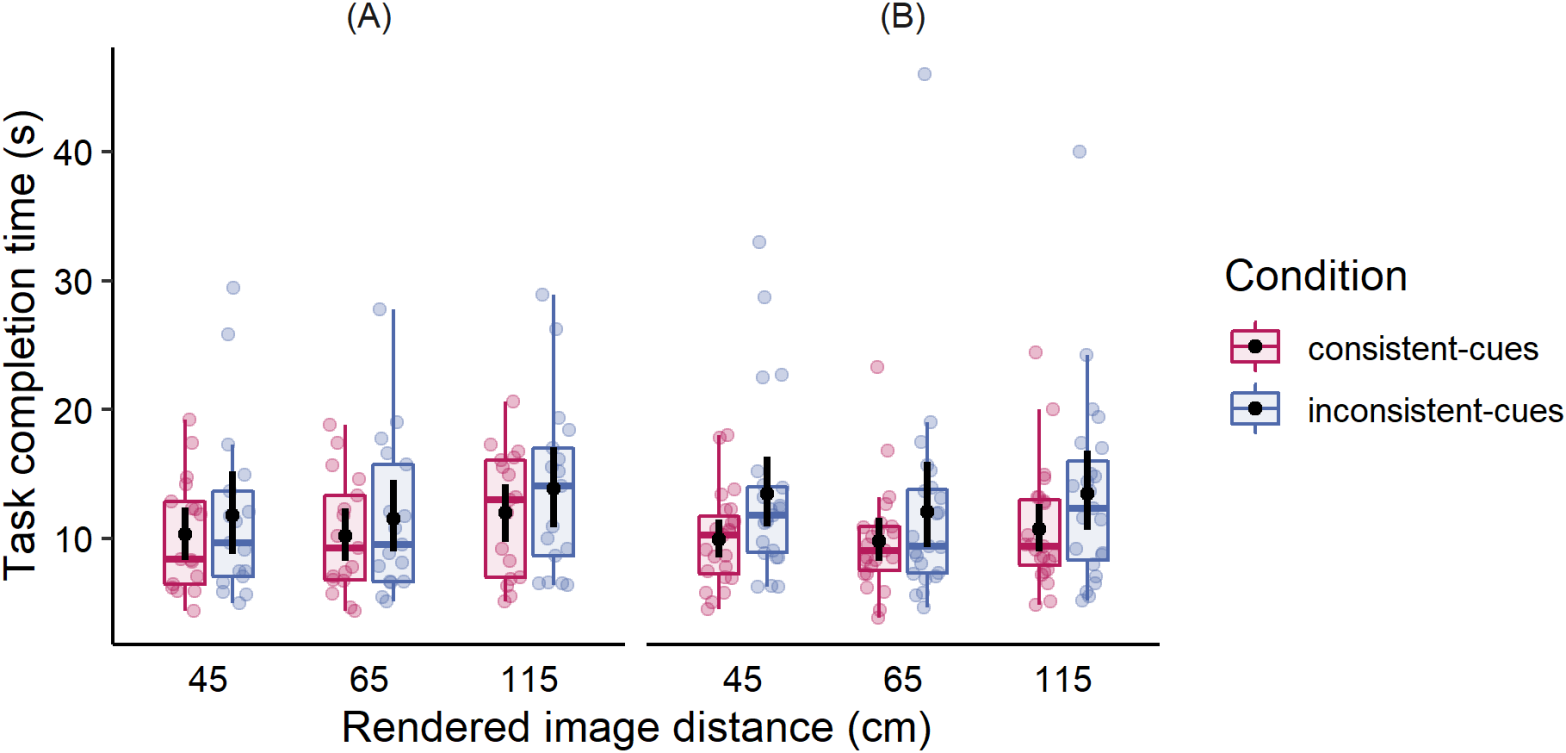
Distance matching task completion time for three rendered image distances in the consistent-cues condition (pink symbols) and inconsistent-cues condition (blue symbols) in two groups: (**A**) with normal vision, and (**B**) with mild binocular and accommodative disorders. Black dots and error bars indicate means and 95% confidence intervals (CIs), respectively. Each color dot represents each individual's average of eight trials. On average, the distances were matched faster if the visualization provided consistent binocular and focus cues.

To explore the temporal aspects of distance matching, the task completion time was assessed in addition to the accuracy of spatial performance. Task completion time was measured in seconds from the moment when the participant submitted a response about the number of circles until the moment when the alignment of the physical pointer was finished. The results are summarized in Figure 6. It is seen that both groups of participants completed the perceptual matching tasks faster when images were displayed on the corresponding focal planes. The task completion times slightly increased when images were demonstrated at 115 cm in comparison to 45 cm distance from the observer, however, no reliable effect was observed. The results of statistical analysis of spatial performance data can be found in the Appendix.

## Discussion

The spatial performance in augmented reality is often assessed from the perspective of the utilized visualization system and environmental factors. However, high interindividual variability in user performance indicates that not everything depends on the quality of information visualization. To extend the current understanding of user specifics, we set out to test how spatial perception in stereoscopic augmented reality is affected by the technical realization of information visualization taking into account individual differences in vision. The implementation of the physical pointer has allowed us to assess the spatial relations between the projected images and the physical object. For the reliable comparison of viewing conditions, an augmented reality display with discrete focal planes was driven in the multifocal and single focal plane mode.

In the field of human-computer interaction, there is an ongoing debate about how to improve user experience and performance in augmented reality. As far as augmented reality displays are intended to be used not only for entertainment, but also for professional purposes, the accuracy of spatial judgments is of a particularly high interest (Condino et al., 2020).

According to cue combination rules (Johnston, Cumming, & Landy, 1994; Landy et al., 1995; Jacobs, 2002; Tyler, 2020), the available information sources contribute according to their relative weights to derive a percept of distance (Svarverud, Gilson, & Glennerster, 2010). Such theories would predict that spatial judgments rely strongly on the availability and reliability of cues in the physical environment and digital overlays when the real and virtual worlds co-exist. Consequently, the distances of objects and images should be matched accurately when both environments provide concordant depth cues. Our findings are in line with these predictions as the most accurate judgments about spatial relations between images and the physical object were made when information visualization was ensured on the corresponding focal planes resulting in the consistency of binocular and focus cues. However, the matched distances were not veridical. A possible explanation is the lack of other concordant cues, such as texture and image size, in the demonstrated images as we aimed to investigate the effect of cues consistency related to different means of information visualization in respect to the display focal planes. Previous studies showed that adding a larger number of concordant cues to the displayed images might further improve the accuracy of spatial judgments in augmented reality (Diaz, Walker, Szafir, & Szafir, 2017; Ping, Weng, Liu, & Wang, 2019), accelerate decision making (Mather & Smith, 2004), and even mask the effect of vergence-accommodation conflict on performance accuracy at 1.0–2.5 m viewing distance (Vienne et al., 2020).

It should be noted that the contribution of other cues becomes more important with an increase of viewing distance as the reliability of binocular and focus cues changes. The effectiveness of cues is not homogenous across the visual space. Specifically, both binocular and focus cues are inversely proportional to the viewing distance (Howard & Rogers, 2002). As binocular and focus cues become less reliable with an increase of viewing distance (Johnston, Cumming, & Landy, 1994), choice variability grows leading to a higher uncertainty in decision making. In turn, the difficulty of making a decision on spatial relations between displayed images and physical environment may be reflected in the time needed to complete the perceptual task. However, we did not find strong evidence supporting this assumption, possibly due to task complexity.

The decision uncertainty and mismatch of spatial layout can be amplified when images are presented in a stereoscopic way as the conflict occurs between binocular and focus cues. We expected that the impact of vergence-accommodation conflict would reflect in the impaired performance and increased overestimation of distances as discussed (Drascic & Milgram, 1996; Pelli, 1999) and shown in a number of studies (Swan, Singh, & Ellis, 2015; Lin & Woldegiorgis, 2017; Singh, Ellis, & Swan, 2018; Lin, Caesaron, & Woldegiorgis, 2019). In general, the distance matching was performed slower when the visualization system provided conflicting binocular and focus cues; however, a strong overestimation was not the case for all participants. Crucially, the individuals with mild binocular and accommodative disorders largely overestimated image distances in the presence of conflicting cues, whereas the mismatch direction changed less in individuals with normal vision, possibly meaning that they outperformed in tolerating the vergence-accommodation conflict when determining the spatial layout of the displayed image and the real object. Thus, our study indicated that the impact of visualization method on spatial perception in augmented reality is modulated by the state of the visual system in terms of binocular and accommodative functions.

The impact of image discrepancies on the perceptual state depends on the range of misalignments that the sensorimotor system is able to overcome. To perceive stereoscopic display's images, vergence eye movements align the two visual axes toward the point of fixation. The constant resting point of the vergence controller is known as phoria. In general, phorias are tolerated as long as the misalignment of retinal images can be compensated by fusion. Previous studies have shown that inducing changes in phorias using prisms causes errors in distance judgments (Shandiz, Hoseini-Yazdi, Ehyaei, & Baghebani, 2012; Daniel & Kapoula, 2019) and reduced stereoacuity (Heravian, Yazdi, Ehyaei, Baghebani, Mahjoob, Ostadimoghaddam, Yekta, & Azimi, 2011), however, no correlation has been found between naturally occurring phorias and performance accuracy in participants with normal vision. When a whole set of visual processes is executed appropriately, the person sees a single binocularly fused image. If one of the stages fails, diplopia or monocular suppression will occur (Spiegel, Baldwin, & Hess, 2016).

Previous studies reported ocular signs of visual stress induced by the use of stereoscopic visualization systems (Mon-Williams, Wann, & Rushton, 1993; Wee, Moon, Lee, & Jeon, 2012; Karpicka & Howarth, 2013; Yoon, Kim, Park, & Heo, 2020). Specifically, it was shown that the induced stress resulted in a deficit of binocular stability after only 10 minutes of exposure to images with conflicting binocular and focus cues (Mon-Williams, Wann, & Rushton, 1993). Observed shifts of horizontal phorias (Mon-Williams, Wann, & Rushton, 1993; Karpicka & Howarth, 2013) and altered near point of convergence (Wee et al., 2012; Yoon et al., 2020) might be proposed as indicators of the increased load on the convergent fusional reserves (Wann, Rushton, & Mon-Williams, 1995; Erkelens & Bobier, 2020). Moreover, it was demonstrated that the accuracy of spatial judgments in the stereoscopic environment correlated with convergent fusional reserves, near point of convergence, and stereoscopic acuity thresholds (McIntire et al., 2014), and fusional reserves allowed the prediction of the realism of depth in stereoscopic displays (Hibbard, Haines, & Hornsey, 2017). Our findings also suggest that the assessment of fusional reserves might be helpful in predicting user performance because individuals with comparatively low fusional reserves showed larger differences in distance matching in response to changes in the consistency of binocular and focus cues.

The conflict between different signals is a challenge for the visual system to be tolerated or solved by assessing the reliability of cues. Generally, individuals demonstrate a high tolerance to blur (Horwood & Riddell, 2014; Horwood, 2019). Consequently, the combination of depth cues can be weighted heavily in favor of binocular cues in the assessment of performance using the cue conflict paradigm (Swenson, 1932; Mather & Smith, 2000; Vienne et al., 2018; Daniel & Kapoula, 2019). In our study, that might explain why distance matching could be less altered by cues consistency in participants with normal vision and fusional reserves allowing individuals to cope with the induced binocular stress and more successfully align the physical object with the displayed images. However, the dissociation of vergence and accommodation may occur not only due to the stereoscopic visualization, but also due to the inability to accommodate or converge properly (Swenson, 1932). For this reason, individuals with reduced visual capabilities can be more susceptible to the visually demanding situation, such as viewing images with conflicting binocular and focus cues. An open question that remains is how exactly cue combination is modulated by binocular and accommodative anomalies.

Despite that the function of vergence or accommodation is modified the most, it leads to the imbalance of the entire system. For this reason, the common situation is that both accommodation and vergence functions are affected and changed to some extent. We suggest that the imbalance of binocular and accommodative systems can result in an increased variability of cues which in turn should alter the accuracy of judgments on three-dimensional spatial locations according to cue combination models. It should be noted that the mechanisms underlying differences in cue weighting are necessarily a matter of speculation at this point, as we did not measure the vergence and accommodation response.

Over recent years, the interest toward investigating binocular and accommodative disorders has increased revealing that many cases of imbalance in the visual functions remain undiagnosed or underdiagnosed due to different reasons (Cacho-Martínez, García-Muñoz, & Ruiz-Cantero, 2010; Paniccia & Ayala, 2015; Hussaindeen, Rakshit, Singh, George, Swaminathan, Kapur, Scheiman, & Ramani, 2017; Magdalene, Dutta, Choudhury, Deshmukh, & Gupta, 2017; Atiya et al., 2020). First, the patients may have no symptoms or complaints. This is a usual situation if the anomalies are mild. Second, there are still no comprehensive assessment criteria to set a diagnosis. Namely, the parameters of anomalies vary considerably both in clinical practice and research. Overall, as binocular and accommodative disorders may affect learning abilities, work efficiency, and quality of life, the corresponding research has become especially important.

Our study indicated the relevance of this issue to the development of augmented reality displays. For perceptual studies, it is important to take into account that the state of vision may contribute to the results when testing a new visualization system. Specifically, we have shown that individuals with decreased binocular and accommodative function are more sensitive to the changes of the interposition of focal planes and image planes in stereoscopic augmented reality, which is reflected in the accuracy of distance matching and magnitude of overestimation. This finding contributes to the existing knowledge and allows us to make important suggestions for further studies. Traditionally, the vision of participants was checked with a limited number of tests assessing only visual acuity and stereoscopic acuity (Napieralski et al., 2011; Kytö, Mäkinen, Tossavainen, & Oittinen, 2014; Singh, Ellis, & Swan, 2018). Sometimes participants were asked to report about the quality of their vision (Swan et al., 2006; Lin & Woldegiorgis, 2017; Lin, Caesaron, & Woldegiorgis, 2019). As has been mentioned, the problem is that individuals may have binocular and accommodative disorders, but normal visual acuity and stereoscopic acuity, as well as no symptomatic complaints. Therefore, we assume that vague inclusion criteria could lead to the participation of individuals with binocular and accommodative disorders along with those who have normal vision. That, in turn, would explain the nonuniformity of responses when assessing spatial perception. If future studies aim to have a homogenous group in respect to binocular and accommodative functions, a rigorous vision screening should be performed. It is worth noting that a high prevalence of vision disorders may lead to the exclusion of most recruited participants (Horwood & Riddell, 2008).

In our study, all participants were asymptomatic, however, the results of the vision screening elucidated the presence of mild binocular and accommodative disorders in most of them. This is in line with the latest reports on the prevalence of binocular and accommodative disorders among the population (Paniccia & Ayala, 2015; Hussaindeen et al., 2017; Magdalene et al., 2017; Atiya et al., 2020). Namely, it was estimated that the non-strabismic binocular vision anomalies are present in at least one third of the young population (Cacho-Martínez et al., 2010; Hussaindeen et al., 2017; Magdalene et al., 2017; Atiya et al., 2020), and prevalence increases with age (Hussaindeen et al., 2017). Accommodative anomalies are even more widespread – reaching nearly two-thirds of population (Cacho-Martínez et al., 2010; Paniccia & Ayala, 2015). Many cases remain undiagnosed or underdiagnosed for several reasons. In particular, patients usually do not exhibit any symptoms or complaints when the anomalies are mild, as the visual system does not experience notable stress in everyday viewing conditions.

Interestingly, the performance variations as a reaction to vergence-accommodation conflict can be amplified depending on the perceptual preferences of depth cues. It was shown that individuals had different preferences for depth cues to rely on (Girshick & Banks, 2009; Wismeijer et al., 2010). Regarding the impact of vergence-accommodation conflict, Horwood and Riddell (2008) suggested that the subject could be classified as a “disparity person” or a “blur person.” The spatial performance of “disparity person” relies on motor fusion (binocular cues), and, therefore, is expected to be less affected by conflicting focus cues. However, the user experience and performance will be strongly degraded for the “blur person” if visualization will provide inconsistent binocular and focus cues. As far as cue preferences are not always linked to the functionality of visual system (Horwood & Riddell, 2008), performance can be affected to different extents in the presence of binocular and accommodative disorders. We did not aim to elucidate the correlation between the spatial judgments in stereoscopic augmented reality and specific vision diagnosis. However, it would be a meaningful direction for future work. Namely, as far as we found differences in two groups even when the reduction in visual functions was mild, it would be worthwhile to continue the investigation to form an understanding of how severe imbalance in the visual system affects the spatial performance and user acceptance of augmented reality displays. Future studies should prioritize the assessment of performance at closer viewing distances as that might be the most indicative in terms of the reaction of the visual system to differences in visualization types, as well as related to the specifics of viewing conditions in professional areas. More research is necessary to synthesize findings from studies on human factors in augmented reality and clinical investigations of vision.

The cue conflict paradigm is often used to study spatial perception and assess modern visualization methods. Monocular suppression and diplopia must be controlled during any experiment in which the vergence-accommodation conflict is present due to the possibility of inducing visual stress. When the binocular function is challenged by the discrepancy of images provided separately for both eyes, a fusion can break resulting in diplopic and blurry images or binocular rivalry with monocular suppression. From a subjective point, it can be accompanied by viewing discomfort, however, it is not always the case. Of the two conditions, suppression is the most difficult to control. Monocular viewing can be dismissed if images for both eyes do not contain specific elements the disappearance of which would be noticed effortlessly. Thus, neither the participant, nor the experimenter would be aware of the suppression manifestation that occurred during the perceptual task. Monocular spatial judgments can differ from binocular ones in the stereoscopic environment (Rosales et al., 2019). It is important to note that the suppression control should be enabled during the entire experiment to be sure that the tasks were completed in the binocular viewing condition. We observed that for some individuals the fusion break did not appear in the very beginning of the task. However, after some time of being challenged, the binocular fusion failed. In this study, we introduced a feature of suppression control that may easily be implemented in the design of visual stimulus. Thus, we emphasize that it is important to include not only the stimulus for binocular fusion (same elements for both eyes), but also the stimulus for the suppression control (different elements for both eyes).

Nowadays, different display architectures ranging from multifocal and varifocal to light field and holographic (Love et al., 2009; Hu & Hua, 2014; Chang, Kumar, & Sankaranarayanan, 2018; Huang & Hua, 2018; Zabels et al., 2019; Chan, Bang, Wetzstein, Lee, & Gao, 2020; Zhan et al., 2020) are proposed to provide the better correspondence of binocular and focus cues. Considering the specifics of human vision, it is important to understand that the correct focus cues may not result in veridical percepts of spatial relations between objects and images in augmented reality. However, the load on the binocular fusion system can be reduced by projecting images on the corresponding focal planes, thus, making the technologies more inclusive from a human-centric perspective. The further improvements of performance accuracy might be achieved by means of corrective feedback and practice (Swan, Singh, & Ellis, 2015; Schmidt, Bruder, & Steinicke, 2017; Rousset et al., 2018; Gagnon, Na, Heiner, Stefanucci, Creem-Regehr, & Bodenheimer, 2020). Therefore, the development of meaningful training can further accelerate the acceptance of new displays.

## Conclusions

We have shown that the consistent-cues method of information representation using a stereoscopic see-through head-mounted display facilitates completion of perceptual tasks in augmented reality. Moreover, our study has shown that attention should be paid to the detailed evaluation of visual functions that would allow for the prediction of the extent of user gain. Specifically, individuals with binocular and accommodative disorders may benefit more from the implementation of multifocal architecture in the head-mounted display in comparison to individuals with normal vision. However, if there is no possibility to check binocular and accommodative functions, it is worth remembering that a patient may not exhibit any symptoms and complaints even when the functionality of the sensorimotor system is not according to the clinical norms. Overall, development of a visualization system that reduces visual stress should be a priority for the successful implementation of augmented reality displays.

## Appendix

We used a Wilcoxon rank-sum test to determine in which visual screening variables participants with mild binocular and accommodative disorders had stochastically different values than participants with normal vision, as most of these variables were strictly non-normally distributed. The results are summarized in Table 1.

**Table 1.** The results of statistical analysis of vision screening outcomes compared between individuals with normal vision and those with mild binocular and accommodative disorders.

Variable	Mdn. dif.	*W*	*p* value
Binocular accommodative facility	3	285.5	0.014
Vergence facility	4	286.5	0.013
Near point of convergence	—	211.5	0.664
Convergent fusional reserves at near	5	275	0.027
Convergent fusional reserves at far	5	242.5	0.049
Divergent fusional reserves at near	2	268.5	0.039
Divergent fusional reserves at far	4	315.5	0.001
Near horizontal phorias	4	105	0.013
Far horizontal phorias	2	107.5	0.012

Spatial performance data showing how accurately and fast participants matched the distance of the demonstrated stimulus were analyzed. The experiment included three independent variables: cues consistency condition (consistent-cues and inconsistent-cues), rendered image distance (45 cm, 65 cm, and 115 cm), and binary variable indicating participants with mild binocular and accommodative disorders. Cues’ consistency condition and rendered image distance were within-subjects variables. Binocular and accommodative disorders’ indicator (bin-acc disorders) was a between-subjects variable.

We were interested in the effects of independent variables on three outcome variables (absolute errors, signed errors, and task completion times). Because measurements were clustered within participants, we used mixed-effects models to assess the influence of independent variables. Intercepts for participants were used as a random effect, whereas all three independent variables were entered as fixed effects into the model. To keep models interpretable, interactions between fixed effects were included.

We used a linear mixed-effect model which was estimated in R (R Core Team, 2020) using the *lmer* function from the *lme4* package (Bates, Mächler, Bolker, & Walker., 2015). The model was fit using maximum likelihood. Model fit quality was evaluated by visually inspecting the normality of residuals. We reported estimated coefficients of fixed effects together with bootstrapped 95% confidence intervals (10,000 bootstrap samples) and *p* values. Reported *p* values for linear model were estimated via *t*-tests using the Satterthwaite approximation to calculate the degrees of freedom. Beta coefficients represent the estimated effects of variables with respect to a reference group (intercept), which are implied by the levels of variables included in the model. For the reference group, we have chosen the results of individuals with normal vision for images displayed at 45 cm distance from the observer in the consistent-cues condition. The results are summarized in Table 2.

**Table 2.** The results of statistical analysis of distance matching outcomes.

	Absolute error			Signed error			Task completion time		
Variable	Beta	95% CI	*p* value	Beta	95% CI	*p* value	Beta	95% CI	*p* value
Intercept	0.08	(0.05, 0.12)	<0.001	0.03	(−0.07, 0.01)	0.135	10.49	(7.92, 13.11)	<0.001
Inconsistent cues	0.04	(0.01, 0.08)	0.025	0.05	(−0.09, −0.01)	0.017	1.89	(0.12, 3.61)	0.036
65 cm	−0.04	(−0.07, −0.01)	0.007	−0.04	(0.01, 0.07)	0.023	−0.14	(−1.61, 1.33)	0.852
115 cm	−0.07	(−0.10, −0.04)	<0.001	−0.05	(0.02, 0.09)	0.003	1.15	(−0.33, 2.58)	0.123
Bin-acc disorders	0.03	(−0.01, 0.06)	0.133	0.04	(−0.09, 0.01)	0.090	−0.70	(−4.00, 2.58)	0.676
Inconsistent cues × bin-acc disorders	0.04	(0.00, 0.07)	0.050	0.06	(−0.10, −0.02)	0.002	1.31	(−0.40, 3.00)	0.133

CI, confidence interval.
